# Dr. Hubbard A. Foster

**Published:** 1903-07

**Authors:** 


Dr. Hubbard A. Foster, of Buffalo, died at his home, No. 3 Saint John's Place, June 16, 1903, aged 55 years. He was born at Adrian, Ohio, and in his youth removed to Rockford, Ill. where his preliminary education was obtained. He volunteered his services during the civil war, enlisting in the 132d Illinois Infantry. Soon after his discharge from the army he located at Buffalo, and received the degree of doctor of medicine from

Harvard Medical School in 1871. Dr. Foster, in 1873, married Florence A. Jenkins, of Bridgewater, Mass. His widow and two children, Florence M. and Henry H., survive. The remains were interred at Clifton Springs, N. Y.

At a meeting of the staff of the homeopathic hospital, of which Dr. Foster had been a member, appropriate resolutions were passed expressing regret at his decease and sympathy with his surviving relatives.

